# Prevention and Control Are Not a Regional Matter: A Spatial Correlation and Molecular Linkage Analysis Based on Newly Reported HIV/AIDS Patients in 2021 in Jiangsu, China

**DOI:** 10.3390/v15102053

**Published:** 2023-10-06

**Authors:** Defu Yuan, Shanshan Liu, Fei Ouyang, Wei Ai, Lingen Shi, Xiaoyan Liu, Tao Qiu, Ying Zhou, Bei Wang

**Affiliations:** 1 Key Laboratory of Environmental Medicine Engineering of Ministry of Education, Department of Epidemiology and Health Statistics, School of Public Health, Southeast University, Nanjing 210009, China; 230239083@seu.edu.cn (D.Y.); 220223703@seu.edu.cn (S.L.); 220213961@seu.edu.cn (F.O.); 2 School of Public Health, Nanjing Medical University, Nanjing 211166, China; 2021110367@stu.njmu.edu.cn; 3 Department of HIV/STD Control and Prevention, Jiangsu Provincial Center for Disease Control and Prevention, Nanjing 210009, China; shilingen@jscdc.cn (L.S.); liuxy@jscdc.cn (X.L.); qiutao@jscdc.cn (T.Q.)

**Keywords:** human immunodeficiency virus, AIDS, spatial analysis, molecular transmission network

## Abstract

HIV-related spatial analysis studies in China are relatively few, and Jiangsu Province has not reported the relevant data in recent years. To describe the spatial distribution and molecular linkage characteristics of HIV-infected patients, this article combined descriptive epidemiology, spatial analysis, and molecular epidemiology methods to analyze patient reporting, patient mobility information, and HIV sequence information simultaneously. The results showed that HIV reporting profiles differed among Jiangsu cities, with the reporting rate in southern Jiangsu being above average. There was a spatial autocorrelation (Global Moran I = 0.5426, *p* < 0.05), with Chang Zhou showing a High–High aggregation pattern. Chang Zhou and Wu Xi were identified as hotspots for HIV reporting and access to molecular transmission networks. Some infected individuals still showed cross-city or even cross-province mobility after diagnosis, and three were linked with individuals in the destination cities within the largest molecular transmission cluster, involving 196 patients. The cross-city or cross-province mobility of patients may result in a potential HIV transmission risk, suggesting that combining timely social network surveys, building an extensive transmission network across cities and provinces, and taking critical regions and key populations as entry points could contribute to improved prevention and control efficiency and promote achievement of the 95-95-95 target and cascade.

## 1. Introduction

In 2022, there were around 1.3 million new HIV infections globally. The 95-95-95 testing and treatment cascade, which aims to ensure that 95% of people living with HIV know their HIV status, 95% of all people living with HIV are accessing treatment, and 95% of all people living with HIV have suppressed viral loads, had been achieved at rates of 86%, 76%, and 71% [1]. These rates were slightly lower in Asia and the Pacific region, including China, at 78%, 65%, and 62% [2], respectively. Despite previous surveys indicating that Yunnan, Sichuan, and Chongqing were the areas most severely affected by the HIV epidemic in China [3], Jiangsu Province, which is located in the eastern region of China, has reported a high level of new cases due to its developed economy, convenient transportation, large jurisdictional span, proximity to several neighboring provinces, and frequent movement of people. From January to October 2022, Jiangsu reported 3677 new HIV cases [4], a decrease of only 2.1% compared with the same period in the previous year [5], and lower than the 16.7% national decline reported at the 8th National Academic AIDS Conference.

HIV, similar to other infectious diseases, exhibits spatial distribution characteristics that are closely related to geographical factors, affecting both its transmission and prevalence. However, previous studies have mainly focused on descriptive analysis of epidemic trends or infection characteristics from a temporal perspective, neglecting the spatial information and failing to comprehensively understand the disease and its impact on different regions and populations. Spatial analysis can fully utilize the spatial information in disease data, so it is widely used in describing the spatial distribution characteristics and changing trends of diseases, and in disease surveillance, evaluation of intervention effects, and exploratory analysis of influencing factors [6,7,8].

The HIV transmission network can be categorized into social and molecular networks. The social network includes confirmed HIV-infected persons, undiagnosed HIV-infected persons, and high-risk contacts without HIV infection. However, constructing a social network can be challenging because identifying the core members is difficult and the individuals and locations where high-risk behaviors occur are relatively concealed. Using the genetic similarity of HIV sequences, a molecular transmission network can be built to investigate transmission patterns, accurately determine potential transmission, and identify active transmission clusters [9]. However, it should be noted that the molecular network only contains HIV-infected individuals and the linkage obtained by genetic distance calculation does not represent the closeness of the transmission relationship and the direction of virus transmission. Therefore, it must be judged in conjunction with the contact history between hosts.

Reports of HIV-related spatial analysis in China are relatively few and mainly focus on a single province, city, or population in the southwestern region of China; insufficient attention has been paid to reporting differences among cities under the same province and the flow of patients during treatment under sub-jurisdictions. Jiangsu Province has also not reported the relevant data in recent years. This paper combined descriptive epidemiology, spatial analysis, and molecular epidemiology methods to analyze the patient reporting data, patient flow information, and HIV sequence information of Jiangsu Province in 2021 to further dissect the spatial distribution characteristics of HIV-infected patients, identify critical areas, and detect molecular linkage of strains between regions. This provided ideas and a scientific basis for developing targeted prevention and control strategies, precise containment of the local HIV epidemic, optimal allocation of health resources, and achieving the 95-95-95 target and cascade.

## 2. Materials and Methods

### 2.1. Data Sources

The data used in this study consisted of two main parts: one from the database of newly reported patients with HIV/AIDS in Jiangsu Province, which included information on the patient’s reporting area and the patient’s address at the first treatment follow up, in addition to essential sociodemographic characteristics; the other part was the HIV sequence information from patients newly reported in 2021 obtained from the laboratory. In addition, information such as the number of residents in each city in Jiangsu Province was obtained from the websites of the provincial and municipal statistical bureaus (Supplementary Material); the maps used were obtained from the website DataV.GeoAtlas (http://datav.aliyun.com/portal/school/atlas/area_selector, accessed on 10 May 2023).

### 2.2. Spatial Analysis

In the spatial analysis, the Moran I index and the Getis-Ord Gi index were calculated using the *spdep* package, and the local indicators of spatial association (LISA) clustering and cold–hot spot maps were drawn using the *ggplot2* package in R. The global Moran I index takes values ranging from −1 to 1 (α = 0.05). When the global Moran I is positive, it indicates a positive correlation in spatial data, which means similar values are more likely to cluster together in space. When the global Moran I index is negative, there is a negative correlation in spatial data, which means that similar values are more likely to be scattered in various regions in space. When the global Moran I index is 0, it indicates no autocorrelation in the spatial data, and the distribution of the spatial data is random. Based on the local Moran I index, regions can be classified into five states: Insignificant, HH (High–High), LL (Low–Low), HL (High–Low), and LH (Low–High), and plotted in the LISA clustering maps. For example, if the value for a region is higher than the values for all the surrounding regions, and the surrounding regions are also all high value regions, this region will be classified as an HH region, and the other states are similarly assigned. The local Getis-Ord Gi* index depends on the difference between the observed and expected values of the spatial data. Hot and cold spots with different confidence intervals (CI) can be identified and marked on the maps. Hot spots indicate that the region has high observed and low expected values, so the disease exhibits significant aggregation in this region, and cold spots represent the opposite.

### 2.3. Laboratory Examination and Molecular Transmission Network Construction

Sample collection, experimental manipulation, and sequence processing have been described in a previous study [10]. According to the Technical Guide for HIV Transmission Networks Monitoring and Intervention (2021 trial version) published by the Chinese Center for Disease Control and Prevention, the phylogenetic tree was first constructed using Fasttree (version: 1.4.3), and the number of clusters and edges under different thresholds was calculated using ClusterPicker (version: 1.2.5) and Hyphy (version: 2.2.4) software, respectively, combining the information from both to select the optimal gene distance threshold (Supplementary Material Figure S1) and complete the visualization of the molecular transmission network using Cytoscape software (version: 3.9.1).

### 2.4. Statistical Analysis

Statistical analysis in this study was mainly performed using R software (version: 4.2.3), including the data frequency and percentage calculations, and image plots.

## 3. Results

### 3.1. Characterization of Newly Reported HIV/AIDS Patient Profiles in 2021

**Figure 1:**
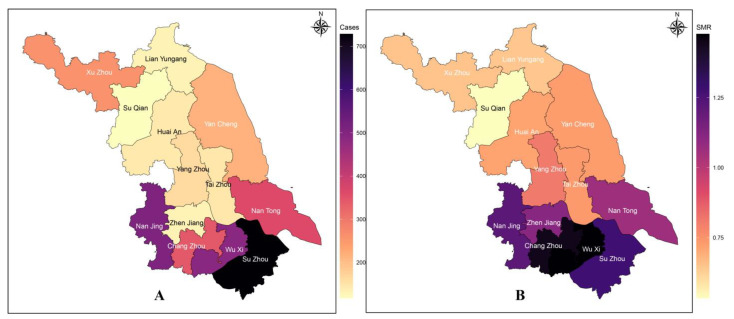
Disease map of the number of newly reported HIV/AIDS patients (**A**) and the standardized reporting rate (**B**) in Jiangsu Province in 2021.

The numbers of newly reported patients with HIV/AIDS in Jiangsu Province (Figure 1), listed by city name from high to low, were Su Zhou, Nan Jing, Wu Xi, Nan Tong, Chang Zhou, Xu Zhou, Yan Cheng, Yang Zhou, Tai Zhou, Huai An, Zhen Jiang, Lian Yungang, and Su Qian. After calculating the standardized reporting rate by combining the number of residents, the standardized rates were ranked Wu Xi, Chang Zhou, Su Zhou, Nan Jing, Zhen Jiang, Nan Tong, Yang Zhou, Tai Zhou, Yan Cheng, Huai An, Xu Zhou, Lian Yungang, and Su Qian from highest to lowest.

**Figure 2:**
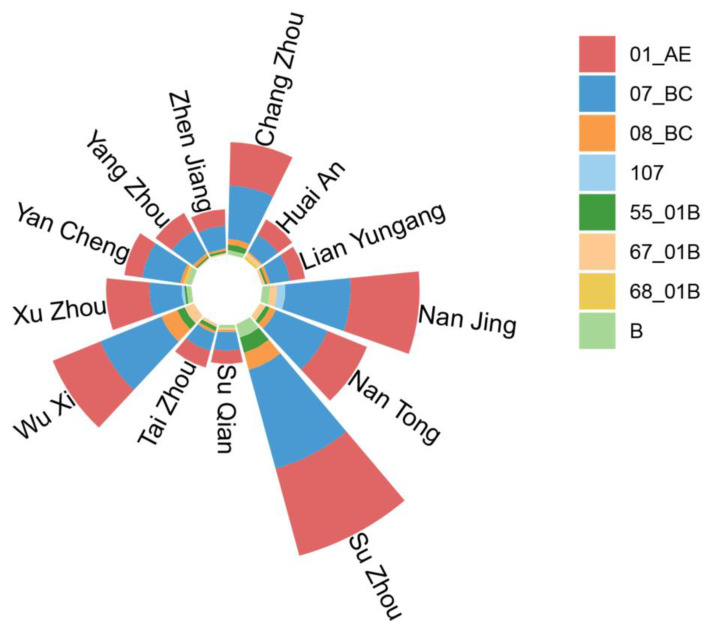
Composition of the top five HIV-1 subtypes in 13 cities in Jiangsu Province.

The composition of the top five HIV-1 subtypes in each city was almost the same (Figure 2), involving eight subtypes, among which Nan Tong, Tai Zhou, Wu Xi, Yang Zhou, Lian Yungang, and Zhen Jiang were dominated by CRF (Circulating Recombinant Form) 01_AE, 07_BC, 08_BC, 55_01B and 67_01B; B, 01_AE, 07_BC, 08_BC and 55_01B dominated in Chang Zhou and Su Zhou. The top five subtypes in Nan Jing and Xu Zhou also included (Unique Recombinant Form) URF_107, while 68_01B reached the top five in Huai An and Yan Cheng.

### 3.2. Spatial Analysis of New Reporting of Patients with HIV/AIDS in 2021

**Figure 3:**
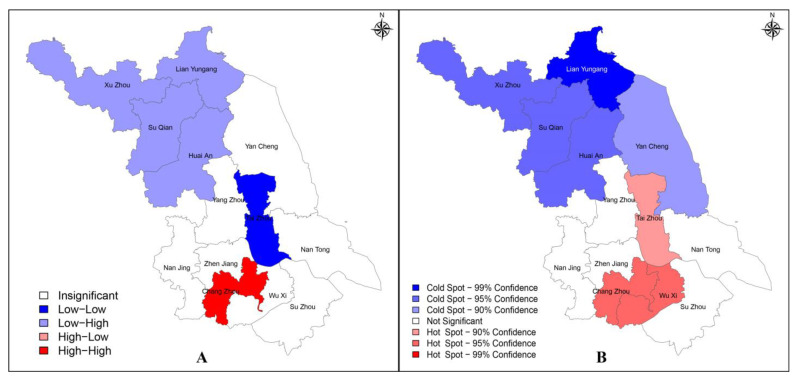
LISA clusters map (**A**) and cold–hot spots map (**B**) of standardized HIV/AIDS reporting rate in Jiangsu Province in 2021.

To identify whether new HIV/AIDS reporting in Jiangsu Province was spatially linked among regions, a spatial analysis was performed using each region’s standardized reporting rate, and a global Moran I = 0.5426 (*p* < 0.05) was obtained, suggesting a spatial correlation. However, this result only suggested the existence of spatial aggregation and did not account for the specific situation of each region, so the local Moran I index and local Gi* index were further calculated. The LISA clustering map (Figure 3A) and the cold–hot spots map (Figure 3B) were drawn based on the corresponding results.

As can be seen from Figure 3, Chang Zhou showed a High–High clustering pattern, Tai Zhou showed a Low–Low clustering pattern, and Xu Zhou, Su Qian, Huai An, and Lian Yungang showed a Low–High clustering pattern. At the 99% CI, only one cold spot was identified in Lian Yungang; at the 95% CI, Chang Zhou and Wu Xi were identified as hot spots, while Xu Zhou, Su Qian, and Huai An were identified as cold spots; at the 90% CI, Tai Zhou and Yan Cheng were identified as hot and cold spots, respectively.

### 3.3. Flow Profiles of Patients between Different Regions in Jiangsu Province at the Time of Reporting and Follow Up

**Figure 4:**
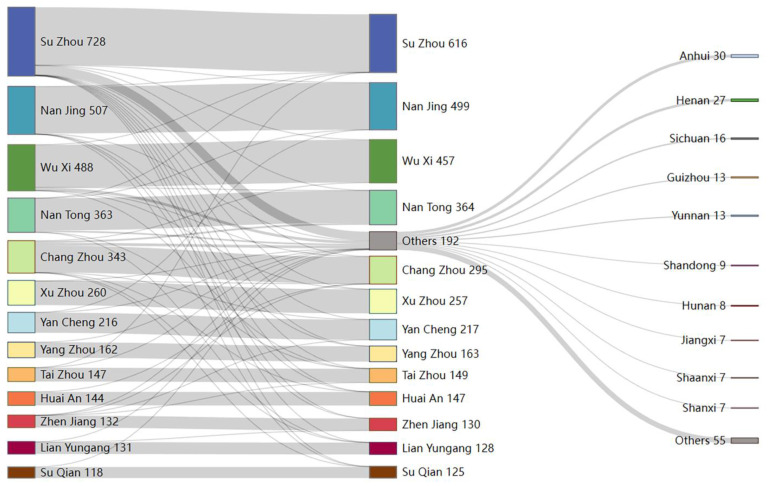
Sankey diagram of permanent address mobility of newly reported HIV/AIDS patients in Jiangsu Province, 2021. Note: Sankey diagrams were constructed using the address of the patient at the time of reporting (first column) and the address of the patient’s usual residence at the first follow up visit after receiving ART (second column) as the coordinates before and after the cross-regional flow of patients, where “others 192” indicates all patients moving outside Jiangsu Province, and the third column shows the top 10 provinces in “others 192”.

The spatial analysis provided autocorrelation within the spatial data and correlation between a region and adjacent regions. However, it could not determine the correlation between non-adjacent regions, yet patient mobility was not limited to adjacent regions. Although cross-regional mobility of patients before reporting of HIV infection was not available, we conducted a mobility analysis using patients’ address areas at the time of reporting and first follow up after antiretroviral therapy (ART) as patient coordinates (Figure 4). The results showed that Su Zhou had the highest number of “destination cities” for patients, with destinations including all 12 cities except Su Zhou. Meanwhile, 116 patients in Su Zhou were reported to have moved to other areas, mainly outside Jiangsu province, while Chang Zhou, Wu Xi, and Su Qian dominated Jiangsu Province. Nan Jing, Wu Xi, Nan Tong, and Tai Zhou each had one patient moving to Su Zhou.

Although patients from Chang Zhou moved to only six cities, Chang Zhou had the highest outflow (thirty patients), among whom twenty went to Nan Tong, four went to Su Qian, three went to Wu Xi, and one each went to Yan Chen, Huai An, and Lian Yungang; meanwhile, Chang Zhou had the highest number of source cities for inflow patients, with five and three patients from Su Zhou and Wu Xi, and the remaining three patients from Xu Zhou, Huai An, and Zhen Jiang, respectively.

Besides Su Zhou and Chang Zhou, Wu Xi, Nan Jing, Xu Zhou, Zhen Jiang, and Lian Yungang also had fewer patients than originally reported because the outflow patients exceeded the inflow patients. In contrast, the treated patients in the remaining six cities were more numerous than the reported patients, with Yang Zhou and Su Qian having only one outflow patient who moved outside the province, and treated patients increased due to the inflow of patients from Su Zhou and Nan Jing, and four patients moving from Chang Zhou to Su Qian.

Except for Huai An, from which only 1 patient went to Chang Zhou after diagnosis, the remaining 12 cities reported 192 patients moving out of the province, involving 28 of the 32 provincial administrative regions in mainland China, and the main destination provinces were Anhui, Henan, Sichuan, Guangzhou, and Yunnan.

### 3.4. Spatial Analysis Based on the Molecular Transmission Network

**Figure 5:**
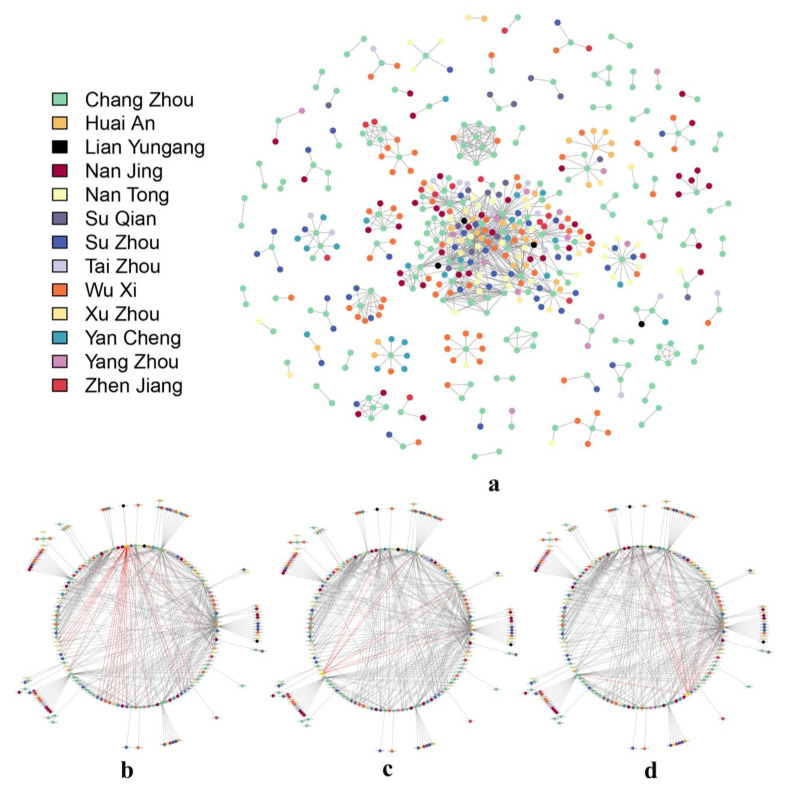
Molecular transmission network of newly reported HIV/AIDS patients in Jiangsu Province in 2021. Note: (**a**): Molecular transmission network based on 3579 pol sequences; the colors of the nodes in the figure corresponds to different regions. (**b**): A patient reported by Chang Zhou, whose usual residence at the first follow up was reported as Huai An. (**c**): A patient reported by Huai An, whose usual residence at the first follow up was reported as Chang Zhou. (**d**): A patient reported by Su Zhou, whose usual residence at the first follow up was reported as Chang Zhou.

The mobility analysis provided clues to the spread of HIV strains between regions. To determine whether there were linkages between the prevalent HIV strains in the regions in which people moved around each other, a molecular transmission network was established based on 3579 pol sequences (Figure 5a). Of these, 501 sequences formed a transmission association with at least one other sequence, based on the criteria of gene distance ≤ 0.02 and Bootstraps ≥ 95%, and were entered into the transmission network analysis, accounting for 14.00% (501/3579) of all sequences. The 501 sequences formed 79 transmission clusters, and the number of sequences included in the clusters ranged from 2 to 196.

The distribution of different regions is shown in Figure 5a. The molecular transmission networks contained sequences from all 13 cities in Jiangsu Province, with sequences from Chang Zhou accounting for the highest proportion of the transmission network (37.33%, 187/501), followed by Wu Xi (14.37%, 72/501) and Su Zhou (8.78%, 44/501), which were also the top three cities in terms of the outflow of newly reported HIV/AIDS patients after diagnosis in Jiangsu Province in 2021.

Most of the molecular transmission networks contained fewer than 10 sequences, mainly formed by sequences from one or two cities. However, 7 of the 79 networks contained more than 10 sequences, and the largest network contained 196 sequences from all 13 cities, with Chang Zhou (22.96%, 45/196) predominating, followed by Wu Xi (12.76%, 25/196), and Nan Tong and Su Zhou tied for third in terms of sequence number (10.20%, 20/196). Of the remaining six networks, five contained sequences at least from Chang Zhou and Wu Xi, and one network consisted of 11 sequences from Chang Zhou, Su Zhou, Tai Zhou, Yan Cheng, and Zhen Jiang.

Eight patients moved between cities during ART in the largest cluster, and three of whom were linked to patients in the destination cities in the largest network. The first patient, reported by Chang Zhou, whose usual residence at the first follow up was reported as Huai An, was linked to 30 other sequences (3 sequences from Huai An) in the molecular transmission network (Figure 5b). The second patient, reported by Huai An, whose usual residence at the first follow up was reported as Chang Zhou, was linked to 12 other sequences (11 sequences from Chang Zhou) in the molecular transmission network (Figure 5c). The third patient, reported by Su Zhou, whose usual residence at the first follow up was reported as Chang Zhou, was linked to 10 other sequences (9 sequences from Chang Zhou) in the molecular transmission network (Figure 5d).

**Figure 6:**
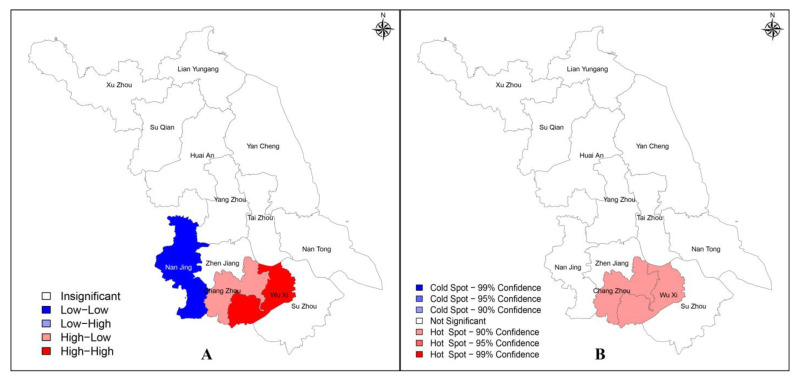
LISA clustering map (**A**) and cold–hot spots map (**B**) of standardized molecular transmission network entry rate in Jiangsu Province in 2021.

Spatial analysis was performed after calculating the standardized network entry rate using the number of entries in each region, and the results showed a global Moran I = 0.5617 (*p* < 0.05), suggesting the existence of spatial autocorrelation, which meant that two adjacent cities in Jiangsu Province tended to have similar network entry rates. LISA clustering maps (Figure 6A) and cold–hot spot maps (Figure 6B) were also drawn using the local Moran I index and local Gi* index. Wu Xi showed a High–High clustering pattern, Chang Zhou showed a High–Low clustering pattern, and Nan Jing showed a Low–Low clustering pattern. Chang Zhou and Wu Xi were identified as hot spots at 90% CI, and no cold spots were identified.

## 4. Discussion

In previous epidemiological studies, the absolute number of HIV infections did not correctly indicate the local HIV infection level. After calculating the standardized reporting rate, this study found that HIV/AIDS patients were unevenly distributed in different cities in Jiangsu, and southern Jiangsu had a higher rate than the average. Spatial analysis revealed that Chang Zhou showed a High–High aggregation pattern and was identified as a hot spot at 95% CI. Patient mobility information also suggested that Chang Zhou ranked first in Jiangsu regarding the number of outflow patients and inflow cities. Meanwhile, the molecular transmission network also showed that some patients who moved between cities were linked with patients from destination cities.

A study in southwestern China noted that urbanization and population density were positively associated with HIV infection [11]. Southern Jiangsu, including Chang Zhou and Wu Xi, occupies the top position in both urbanization and population density in Jiangsu (Table S1 in Supplementary Material), which may be one reason why HIV reporting rates in southern Jiangsu are generally higher than average, and the two adjacent areas of Chang Zhou and Wu Xi are identified as hot spots at 95% CI. However, Su Zhou and Nan Jing, which also have high population density and urbanization rates, were not identified as hot spots. Some studies have pointed out that gross domestic product (GDP) was a protective factor for HIV infection [12,13], and GDP in Su Zhou and Nan Jing far exceeded that of other cities in Jiangsu (Table S1 in Supplementary Material), which may have influenced the occurrence of HIV infection, but further analysis combined with more information is needed.

The survey results in Jiangsu Province from 2009 to 2011 showed that more than half of the HIV-1 genotypes of infected patients were 01_AE, and the main transmission route had changed from heterosexual to homosexual [14]. The survey also reported that 01_AE and 07_BC dominated, and besides subtype B, the rest of the subtypes with a composition ratio of more than 1% were CRFs and URFs in men who have sex with men (MSM) in 2017 and 2018. This study showed that the main subtype composition of newly reported HIV/AIDS patients in 2021 was similar to previous studies but with increased CRFs and URFs. Meanwhile, the proportion of 07_BC exceeded that of 01_AE, which previously ranked first, and the proportion of 08_BC increased from fourth to third [15], suggesting that both the number of HIV-1 subtypes and their proportions in Jiangsu Province were changing. It is necessary to investigate the situation through close and comprehensive surveillance and combine this with monitoring of prevalent HIV-1 strain characteristics to allow timely adjustments of prevention and treatment policies.

Previous studies have shown that the highest prevalence of HIV in Jiangsu Province gradually shifted from Xu Zhou in northern Jiangsu to southern Jiangsu, with Nan Jing, Su Zhou, Wu Xi, Chang Zhou, and other southern areas dominating since 2008, and showing a tendency to radiate to central and northern Jiangsu [16]. This study also found clusters in Chang Zhou and Su Zhou but not in Nan Jing and Wu Xi; it also found clusters in Tai Zhou, which belongs to central Jiangsu, which may be related to the strategies adopted in recent years to strengthen publicity and education, expand the coverage of sentinel sites, and improve the accuracy of counseling and testing in the whole province [4]. The findings suggested that the HIV clustering profiles in Jiangsu changed to a certain extent, and it was necessary to combine the monitoring results in order to direct resources toward high aggregation regions, to carry out prevention and treatment measures such as increasing the frequency of testing for high-risk groups, promote the application of pre-exposure prophylaxis (PrEP), expand the scope of ART, and improve the effectiveness of ART to interrupt the transmission of HIV. This will gradually reduce the number of new HIV infections and ultimately achieve the UNAIDS 2030 GOALS.

The results of this study showed clustering of patients of reported HIV/AIDS in Su Zhou and Chang Zhou, the frequent patient mobility between these two cities, and the widespread presence of sequences from Chang Zhou in different molecular transmission networks, suggesting that HIV-1 strains prevalent in Jiangsu Province were not only spreading within the same city but also spreading among different cities. It is important to note that although three patients in this study had a change of address after diagnosis, and molecular transmission network analysis also revealed that these three patients were linked molecularly to some of the newly reported patients in the destination cities, it was uncertain whether these patients’ movements and molecular linkages were related to the cross-transmission of HIV-1 strains between these cities owing to the lack of information on the patients’ addresses before diagnosis, as well as their mobility characteristics and social transmission networks after infection. Jiangsu Province mainly detects HIV-infected patients through voluntary counseling testing (VCT) clinics [4]. However, active testing based on the perceived HIV infection risk may be more effective than widespread screening methods [17,18]. A study in Jiangsu surveyed the acceptance of smartphone applications for self-testing of HIV infection status among MSM and found that 71.2% of respondents were willing to use this application to increase the testing frequency [19], so multiple testing modes such as self-testing should be developed in addition to testing at health care facilities. Some researchers have pointed out intensive intervention programs in high-risk groups were more efficient in reducing new HIV infections than interventions aimed at the general population [20]. Jiangsu Province has been conducting drug resistance monitoring of all newly reported HIV/AIDS patients since 2017 while continuously expanding the scope of monitoring and improving the monitoring network, as well as cooperating with social organizations to carry out publicity and testing work on high-risk populations to provide conditions for promoting early detection, diagnosis, and treatment of infected patients. At present, Jiangsu is also working on constructing a dynamic molecular transmission network covering all HIV/AIDS patients in the province, hoping to provide critical information for the timely detection of new networks, clarify the transmission characteristics of strains, and deploy prevention and control resources rationally. As a preliminary exploration of network construction, this study needs to be combined with information from epidemiological surveys and social transmission networks to provide directions for further construction.

About 5% of patients moved to other provinces with severe HIV epidemics after diagnosis; they were mainly concentrated in Anhui, Henan, Sichuan, Guizhou, and Yunnan [21]. 85_BC was relatively frequent in Sichuan, in addition to the major subtypes like 07_BC, 01_AE, and 08_BC [22]. The subtype composition was more complex in Yunnan, which borders Myanmar, Vietnam, and Laos, owing to frequent cross-border HIV transmission [23], and the new subtype numbers were significantly higher in Yunnan [24], explaining the difference in HIV-1 subtype composition from Jiangsu. Previous studies have shown that the antibodies produced after HIV infection are insufficient to resist re-infection with other HIV strains [25], and re-infection leads to many adverse outcomes due to the virologic rebound [26]. Movement of patients between cities, and especially across provinces, and failure to maintain optimal ART outcomes and reduce their high-risk behavior, may lead to recombination of different subtypes and new subtypes emerging [27], bringing more challenges to HIV prevention and control. Therefore, realizing timely communication and cooperation among different provinces, focusing on vital mobile populations, jointly improving prevention and control effects, reducing cross-province HIV transmission, and avoiding generation of new CRFs should be the focus of intervention.

This study combined three aspects: patient reporting, patient mobility, and HIV sequence information for the first time in Jiangsu Province to identify cities of high aggregation and mobility as well as active transmission clusters, using spatial and molecular linkages of patients. It has provided reliable information for targeted regional surveillance efforts to strengthen and promote precise interventions. However, there were some limitations of this study: First, this study only included the sequences collected in 2021, and therefore could not identify the changes and linkages of HIV/AIDS patients in each region over time, and only provided clues rather than complete evidence of HIV transmission. Second, although this study identified highly aggregated and high-mobility cities such as Chang Zhou and Wu Xi, and also identified several inter-city flows of patients in the molecular transmission network, further models such as social transmission networks are needed to analyze the role of this frequent mobility of diagnosed patients in HIV transmission, owing to the lack of more information including social, economic, cultural and patient behavioral factors.

## 5. Conclusions

There was a spatial aggregation of newly reported HIV infections in Jiangsu Province in 2021, and there was inter-city mobility of infected individuals after diagnosis. The cities in southern Jiangsu, represented by Chang Zhou and Wu Xi, were critical for epidemic prevention and control. Future studies should combine molecular and social transmission networks to construct a larger transmission network model, taking key regions and key populations as entry points, enhancing prevention and control efforts, improving prevention and control timeliness and efficiency under limited resources, and contributing to achieving the 95-95-95 target and cascade.

## Data Availability

All publicly available data for this study are included in the content, and some data are provided in the Supplementary Materials.

## Supplementary Materials

The following supporting information can be downloaded at: https://www.mdpi.com/article/10.3390/v15102053/s1, Figure S1 Clusters and edges number line graph under different thresholds; Table S1 Characteristics of 13 cities in Jiangsu Province
