# Supplementary material for: Prevention and Control Are Not a Regional Matter: A Spatial Correlation and Molecular Linkage Analysis Based on Newly Reported HIV/AIDS Patients in 2021 in Jiangsu, China
Source: Viruses. 2023 Oct 6;15(10):2053. doi: 10.3390/v15102053 (PMC10612072; doi:10.3390/v15102053)
Supplement: Supplementary file 1 [file viruses-15-02053-s001.zip › viruses-2618730-supplementary.pdf]

Supplementary Materials

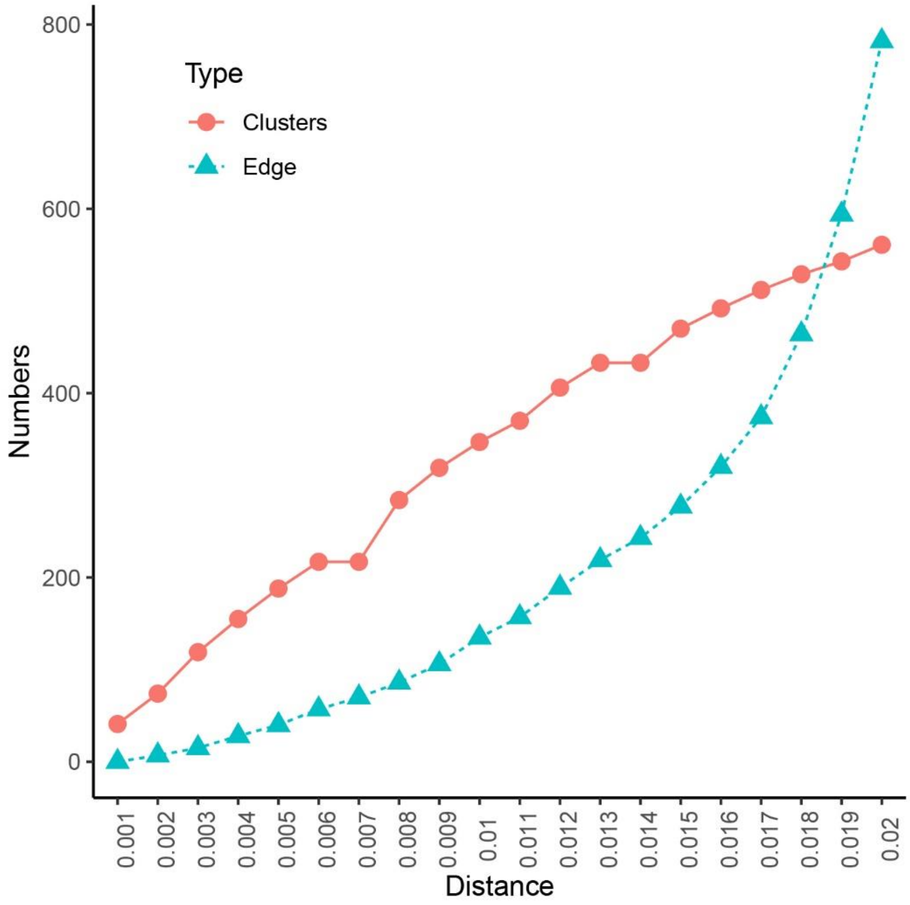

Figure S1 Clusters and edges number line graph under different thresholds

Table S1 Characteristics of 13 cities in Jiangsu Province

Cities	Cases	Permanent resident population	Area (km^2)	Population density (People/km^2)	Urbanization rate (%)
Nan Jing	507	9423400	6587	1430.606	86.8
Wu Xi	488	7464000	4627	1613.140	82.8
Su Zhou	728	12847800	8488	1513.643	81.7
Zhen Jiang	132	2681000	3847	696.907	79.5
Chang Zhou	343	5366200	4372	1227.402	77.1
Yang Zhou	162	4515600	6591	685.116	71.0
Nan Tong	363	7733000	8001	966.504	70.4
Tai Zhou	147	4516800	5787	780.508	68.1
Huai An	144	4562200	10072	452.959	65.7
Xu Zhou	260	9028500	11259	801.892	65.6
Yan Cheng	216	6713000	16972	395.534	64.1
Su Qian	118	4986800	8555	582.911	62.2
Lian Yungang	131	4602000	7615	604.334	61.5
